# Impact of Diabetes Medication Therapy Adherence Clinic (DMTAC) appointment intervals on glycemic control in public health clinics across Perak, Malaysia

**DOI:** 10.51866/oa1341

**Published:** 2022-11-13

**Authors:** Ying Shan Beh, Keshamalini Gopalsamy, Sabrina Lai Fong Lee, V Paranthaman P. Vengadasalam

**Affiliations:** B.Pharm (Hons.) USM, Klinik Kesihatan Greentown, Jalan Raja Musa Aziz, Ipoh, Perak, Malaysia. Email: behyingshan@gmail.com; B.Pharm (Hons.) (USM), Klinik Kesihatan Jalan Oya, Jalan Oya, Sibu, Sarawak, Malaysia.; B.Pharm (Hons.) (USM), Klinik Kesihatan Greentown, Jalan Raja Musa Aziz, Ipoh, Perak, Malaysia.; MBBS (Malaya), M.Med (Family Medicine) Malaya Klinik Kesihatan Greentown, Jalan Raja Musa Aziz, Ipoh, Perak, Malaysia.

**Keywords:** Diabetes, DMTAC, Appointment, Pharmacist

## Abstract

**Introduction:**

Frequent diabetes medication therapy adherence clinic (DMTAC) appointments may lead to more rapid glycaemic control. This study aimed to evaluate the association between appointment intervals and glycaemic control (haemoglobin A1c [HbA1c] level) along with blood pressure (BP) and lipid profile (LP) during DMTAC appointments.

**Method:**

This study retrospectively reviewed all recorded baseline and completed DMTAC data, including HbA1c level, LP and BP, of 318 eligible participants from 29 DMTACs across Perak. The participants were divided into shorter appointment interval (SAI) (≤30 days) and longer appointment interval (LAI) groups.

**Results:**

The majority of the baseline socio-demographic and clinical characteristics did not significantly differ between the SAI and LAI groups (p>0.05). Ischaemic heart disease (Odds ratio, OR=3.457; 95% CI= 1.354-8.826; p=0.009) and hypertension (OR=0.521; 95% CI=0.276-0.992; p=0.044) were significantly associated with the appointment intervals. Upon completion of eight DMTAC visits, the HbA1c and FBS levels and DBP significantly improved (p<0.05). However, the mean HbA1c level (1.35±2.18% vs 0.87±2.11%, p=0.548), FBS level (1.25±4.82mmol/L vs 2.29±6.23mmol/L, p=0.538), SBP (3.28±21.82mmHg vs 3.65±18.35mmHg, p=0.343) and LDL level (0.09±0.98mmol/L vs 0.07±1.13mmol/L, p=0.246) did not significantly differ between the SAI and LAI groups.

**Conclusion:**

Longer DMTAC appointment intervals had similar improvement in glycaemic controls, blood pressure and lipid profiles as compared to shorter appointment intervals. A longer interval can be scheduled for lower-risk patients to optimise the use of human resources and minimise costs.

## Introduction

The International Diabetes Federation Atlas estimated the prevalence of diabetes globally to be 9.3% (463 million people) in 2019. It is expected to increase by 0.9% and 1.6% in 2030 and 2045, respectively.^1^ The 2019 National Health and Morbidity Survey showed that the prevalence of Type 2 Diabetes Mellitus, T2DM had increased in Malaysia from 13.4% in 2015 to 18.3% in 2019.^2^ Therefore, a multidisciplinary approach is crucial for the management of T2DM.^3^

As part of multidisciplinary teams, pharmacists run diabetes medication therapy adherence clinics (DMTACs) in collaboration with physicians since 2004 in Malaysia by optimising the use of medications and providing patient education on disease, adherence and lifestyle.^4^ The involvement of pharmacists following a structured educational module training has been reported to significantly improve medication adherence and glycaemic control.^1,3,5-9^ Interventions are provided at every visit regardless of the interval of visits following a standard guideline of 30-120 days in Malaysia.^8,9^

Regarding visit intervals, frequent clinic appointments every 7-14 days were found to be superior in glycaemic control (p<0.05) in Brazil and the United States.^10,11^ This strategy helps physicians to control clinical inertia by optimising and intensifying diabetes management with multidisciplinary staff.^10^ Furthermore, a more frequent clinic visit could improve medical outcomes and reduce costs on a long-term basis because of markedly fewer visits to the emergency room.^12^ However, no studies have yet to specifically evaluate the impact of DMTAC appointment intervals in achieving the HbA1c level target in Malaysia. Frequent or short-interval clinic visits may be challenging among pharmacists owing to the increasing patient load according to the Annual Report 2017 by the Ministry of Health in Malaysia.^13^

This study primarily aimed to determine the association between glycaemic control (haemoglobin A1c [HbA1c] and fasting blood sugar [FBS] levels) and appointment intervals in DMTACs following a standard practice of 30-day appointment scheduling for partial supply medication collection in pharmacies as a cut-off point. Shorter appointment intervals (SAIs; ≤30-day interval) with frequent clinic visits were compared with longer appointment intervals (LAIs) with fewer clinic visits. In addition, the impact of DMTAC appointment intervals on the lipid profile (LP) and blood pressure (BP) was evaluated.

## Methods

### Study design

In this retrospective observational study, we identified DMTAC patients recruited from January to December 2017 by a pharmacist with ≥1-year experience. The inclusion criteria were as follows: (1) at least eight completed DMTAC visits and (2) availability of clinical data (HbA1c or FBS level, LP and BP) within 6 months pre-recruitment and after the eighth completed visit. The exclusion criteria were as follows: (1) incomplete DMTAC visits, (2) unavailability of clinical data (HbA1c or FBS level, LP and BP) within 6 months prerecruitment and after the eighth completed visit.

### Study clinic

**Figure 1:**
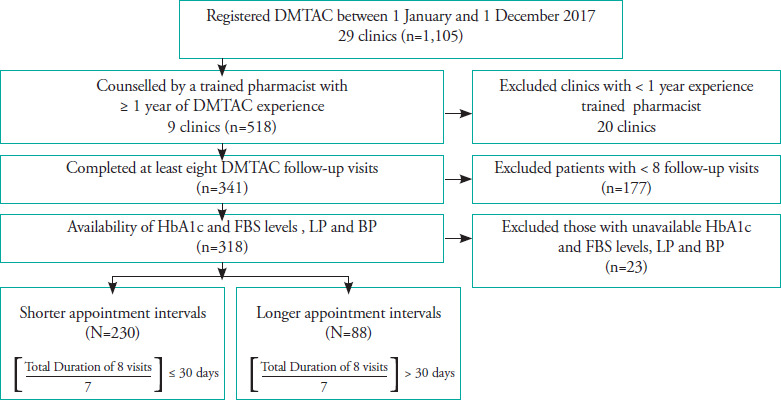
Methodology

Abbreviations: DMTAC, Diabetes medication therapy adherence clinic; HbA1c, glycated haemoglobin; FBS, fasting blood sugar; LP, lipid profile; BP, blood pressure.

A total of 29 established public DMTACs in Perak were included, with a total number of 1,105 newly recruited DMTAC patients from January to December 2017 as shown in **Figure 1**. These clinics have conducted >1 year of DMTAC service, according to the protocol by the Pharmaceutical Services Division, Ministry of Health, Malaysia.^4^ Pharmacists underwent 5–10 days of structured training module before the service. During the visits, patients were recruited following the criteria stated in the protocol: (i) uncontrolled diabetes despite optimum medications and dose prescribed, (ii) non-compliance with medications, (iii) HbA1c level of >8.0% or (iv) co-morbidities, multiple medications or complications (macro-vascular and micro-vascular) with available baseline data on the HbA1c and FBS levels, LP and BP. The patients were required to complete a minimum of eight DMTAC visits. Each intervention during the visit was performed accordingly.^4,14^ Upon completion of the eighth visit, the HbA1c and FBS levels, LP and BP were recorded again.

### Study population

We identified 341 study participants from manual and electronic medical records (Pharmacy Information System) and obtained socio-demographic data, clinical characteristics, appointment intervals, laboratory parameters and medication information.

### Appointment interval

The patients were grouped into the SAI group with frequent clinic visits and LAI group with fewer clinic visits. According to the DMTAC protocol, the number of visits per patient must be ≥8.^4^ Therefore, the participants were grouped by summing the total duration between each appointment and dividing it by 7, as there are a total of seven intervals in between eight visits. The final value was used for grouping.

### Matching

The recruited patients were matched in terms of (a) mean age (<55, 55–64 , 65–74 and ≥75 years) and (b) mean baseline anti-glycaemic regimen (oral hypoglycemic agent [OHA] only, OHA + basal only, OHA + premixed, OHA + basal bolus or insulin only) at baseline between the SAI and LAI groups to reduce discrepancies in the HbA1c level.^15,16^ This is because the HbA1c level target for each person varies. A younger patient’s HbA1c level target is 6.0–6.5%, while an older patient’s target is 7.1–8.0%. Hence, the management varies according to the target. The treatment regimen for each patient also affects the HbA1c level, as each group of OHAs and insulin type have different HbA1c level reduction power.^17^ Therefore, these two factors were included as part of the matching criteria.

### Statistical analysis

Data were analysed using IBM SPSS Statistics 23.0 (IBM SPSS Statistics, IBM Corporation, Armonk, New York). Baseline data were examined using the Chi-square test and independent t-test. Association between the variables and outcome was evaluated using binary logistic regression. During comparison within and between the groups, the mean HbA1c level, FBS level, BP and LDL level were analysed using repeated measures ANOVA. The data were presented as frequencies (percentages), means (standard deviations) or mean differences (95% CIs), as appropriate. For all analyses, a *p-value* of <0.05 was considered statistically significant.

## Results

A total of 318 patients were included in this study and divided into the SAI (mean: 25±5.63 days; n=230) and LAI groups (mean: 50±23.17 days; n=88) according to their mean appointment interval throughout eight visits. As shown in **Table 1a**, the patient age ranged from 25 to 91 years, and the mean age was 59.8±10.3 years. The male-to-female sex ratio was 1:1.4, and majority of the participants were Malays (48.1%), followed by Chinese (27.7%), Indians (23.6%) and others (0.6%). Meanwhile, 16 patients had missing data and hence were excluded from the analysis.

**Table 1a:** Baseline socio-demographic data of the participants.

Variable	Shorter appointment interval group n, (%) 230 (100)	Longer appointment interval n,(%) 88 (100)	*P-value*
*Age (year)*			
<55	72 (31.3)	17(19.3)	0.068^a^
55-64	93 (40.4)	34 (38.6)	
65-74	52 (22.6)	29 (33.0)	
≥75	13 (5.7)	8(9.1)	
*Sex*			
Male	98 (42.6)	34 (38.6)	0.51^a^
Female	132 (57.4)	54 (61.4)	
*Ethnicity*			
Malay	131 (57.0)	22 (25.0)	<0.001^a^
Chinese	45 (19.6)	43 (48.9)	
Indian	53 (23.0)	22 (25.0)	
Others	1 (0.4)	1 (1.1)		

a Chi-square test

**Table 1b:** Baseline clinical characteristics of the participants.

Variable	Shorter appointment interval group n, (%) 230 (100)	Longer appointment interval group n, (%) 88 (100)	*P-value*
*BMI (kg/m^2^) [mean±SD]*	28.06 (6.09)	27.45 (5.71)	0.492^a^
*Blood pressure (mmHg) [mean±SD]*			
Systolic	142.66 (21.67)	137.90 (17.74)	0.081^a^
Diastolic	81.82 (11.06)	77.94 (9.98)	0.007^a^
*Blood sugar profile [mean±SD]*			
FBS (mmol/L)	10.87 (4.46)	10.71 (4.24)	0.792^a^
RBS (mmol/L)	12.15 (5.10)	10.98 (4.68)	0.176^a^
HbAlc (%)	10.61 (2.27)	10.33 (2.05)	0.383^a^
*Lipid profile (mmoUL) [mean±SD]*			
Total cholesterol	5.08 (1.51)	4.79 (1.06)	0.169^a^
Triglyceride	1.95 (1.24)	2.01 (1.18)	0.736^a^
LDL	2.71 (1.09)	2.69 (1.17)	0.971^a^
HDL	1.28 (0.44)	1.48 (1.15)	0.160^a^
*Renal profile [mean±SD]*			
Creatinine (umol/L)	91.54 (51.59)	95.17 (56.96)	0.641
*Type of co-morbidities*			
Ischaemic heart disease	11 (4.8)	13 (14.8)	0.003^b^
Hypertension	184 (80.0)	62 (70.5)	0.069^b^
Dyslipidaemia	136 (59.1)	53 (60.2)	0.859^b^
Chronic kidney disease	22 (9.6)	11 (12.5)	0.443^b^
*Anti-diabetic regimen* ^c^			
OHA only	47 (21.5)	19 (21.8)	
OHA + basal insulin	64 (29.2)	23 (26.4)	
OHA + pre-mixed insulin	40 (18.3)	17 (19.5)	0.997^b^
OHA + basal bolus insulin	49 (22.4)	21 (24.1)	
Insulin only	16 (7.3)	6 (6.9)	

a Independent t-test

b chi-square test

c Sixteen patients had missing data.

All baseline characteristics as shown in **Table 1a** and **1b** did not significantly differ between the SAI and LAI groups (p>0.05), except for ethnicity, diastolic blood pressure (DBP) and ischaemic heart disease (IHD). Variables with p<0.25 were subjected to binary logistic regression analysis as summarised in **Table 2**.

**Table 2:** Binary logistic regression analysis for the association between the baseline variables and appointment intervals.

Variables	OR	95% CI	p-value
**Socio-demographic**			
*Age (year)*			
<55	1		
55–64	1.428	0.688–2.961	0.339
65–74	2.047	0.935–4.482	0.073
≥75	1.775	0.555–5.678	0.334
*Ethnicity*			
Malay	0.127	0.007–2.284	0.162
Chinese	0.624	0.035–11.289	0.750
Indian	0.310	0.017–5.623	0.428
Others	1		
**Clinical characteristics**			
*Blood pressure*			
Systolic	1.022	0.990–1.054	0.176
Diastolic	0.953	0.903–1.006	0.083
*Glycaemic control*			
Random blood sugar	0.965	0.872–1.069	0.497
Variables	OR	95% Cl	p-value
*Co-morbidity: ischaemic heart disease*			
Yes	3.457	1.354-8.826	0.009
*Co-morbidity: hypertension*			
Yes	0.521	0.276-0.982	0.044
*Cholesterol level*			
Total cholesterol	1.398	0.941-2.076	0.097
HDL	1.214	0.701-2.103	0.488

Abbreviations: OR, odds ratio; CI, confidence interval; HDL, high-density lipoprotein

In **Table 3**, HbA1c level, FBS level and DBP significantly differed between baseline and after the eighth visit within the groups.

**Table 3:** Within-group comparison of glycaemic control, BP and LDL level after completing the eight visits using repeated measures ANOVA.

Variables		SAI			LAI		
		Mean (SD)	Mean diff	p-value	Mean (SD)	Mean diff	p-value
*HbAlc*	Baseline	10.16 (1.98)	1.35	<0.001	10.21 (2.14)	1.08	<0.001
	After the eighth visit	8.81 (1.83)			9.13 (1.93)		
FBS	Baseline	10.65 (4.86)	1.25	0.018	10.80 (4.19)	2.29	0.005
	After the eighth visit	9.40 (3.64)			8.51 (5.42)		
SBP	Baseline	140.55 (21.02)	3.28	0.107	138.50 (18.07)	3.65	0.09
	After the eighth visit	137.26 (18.87)			134.85 (31.87)		
DBP	Baseline	81.62 (1.03)	2.39	0.018	78.15 (1.10)	3.49	0.01
	After the eighth visit	79.23 (0.86)			74.66 (1.82)		
LDL	Baseline	2.62 (0.16)	0.15	0.568	2.85 (0.17)	0.07	0.688
	After the eighth visit	2.53 (0.14)			2.78 (0.20)		

Abbreviations: SAI, shorter appointment interval; LAI, longer appointment interval; SD, standard deviation; HbA1c, glycated haemoglobin; FBS, fasting blood sugar; SBP, systolic blood pressure; DBP, diastolic blood pressure; LDL, low-density lipoprotein.

As shown in **Table 4**, the mean HbA1c level (p=0.548), FBS level (p=0.538), systolic blood pressure (SBP) (p=0.343) and LDL level (p=0.246) after the eighth visit did not significantly differ between the SAI and LAI groups. The mean difference in each variable was compared, and the normality of continuous data was determined using the central limit theorem. All the variables used in this study are defined in **Table 5**.

**Table 4:** Main effect of the appointment intervals on glycaemic control, BP and LDL level between the groups using repeated measures ANOVA.

Variable	Group (n)	Mean diff (SD)	*p-value*
Difference in the HbA1c level	SAI (75)	1.35 (2.18)	0.548
	LAI (52)	0.87 (2.11)	
Difference in the FBS level	SAI (86)	1.25 (4.82)	0.538
	LAI (62)	2.29 (6.23)	
Difference in the SBP	SAI (117)	3.28 (21.82)	0.343
	LAI (76)	3.65 (18.35)	
Difference in the DBP	SAI (117)	2.39 (10.75)	0.002
	LAI (74)	3.49 (11.58)	
Difference in the LDL level	SAI (42)	0.09 (0.98)	0.246
	LAI (41)	0.07 (1.13)	

Abbreviations: SAI, shorter appointment interval; LAI, longer appointment interval; SD, standard deviation; HbA1c, glycated haemoglobin; FBS, fasting blood sugar; SBP, systolic blood pressure; DBP, diastolic blood pressure; LDL, low-density lipoprotein.

**Table 5:** Variables used in this study.

Variable	Operational definition	Measuring scale
Age	Age of patient in completed years obtained from medical records	Year
Sex	Sex of patient obtained from medical records	Male Female
Ethnicity	Category of people who share certain inherited physical characteristics obtained from medical records	Malay Chinese Indian Others
Weight	Weight of patient obtained in kilogrammes obtained by staff at the time of clinic visit using a weighing scale	Kilogramme
Height	Height of patient obtained in centimetres obtained by staff at the time of clinic visit using a Seca scale	Centimetre
Blood pressure	Systolic and diastolic blood pressures obtained on the recruitment day and on the eighth visit obtained from medical records	Millimetre of mercury
Fasting blood sugar level	Level of glucose in the blood after an 8-hour fast, measured within 6 months before recruitment and within 6 months after the eighth visit	Millimole/liter
HbAlc level	Glycosylated haemoglobin level showing the average level of blood sugar over the past 2-3 months, measured within 6 months before recruitment and within 6 months after the eighth visit	Percentage
Total cholesterol level	Total amount of cholesterol, measured within 6 months before recruitment and within 6 months after the eighth visit	Millimole/liter
Triglyceride level	Level of an ester formed from glycerol and three fatty acid groups, measured within 6 months before recruitment and within 6 months after the eighth visit	Millimole/liter
LDL level	Level of the principal transporter of cholesterol and fat in human blood, measured within 6 months before recruitment and within 6 months after the eighth visit	Millimole/liter
HDL level	Level of the transporter of cholesterol from the tissues to the liver for excretion, measured within 6 months before recruitment and within 6 months after the eighth visit	Millimole/liter
Creatinine level	Creatinine is a waste product from the normal breakdown of muscle tissue, measured within 6 months before recruitment and within 6 months after the eighth visit	Micromole/liter
Estimated glomerular filtration rate	A measure of the function of the kidneys, calculated on the basis of the amount of creatinine in the blood	Millilitre/minute
Co-morbidities	The presence of one or more additional diseases or disorders co­occurring with T2DM at recruitment and the eighth visit	Yes No
Number of OHA(s)	Number of oral hypoglycaemic agents used obtained on the recruitment day and the eighth visit obtained from medical records	1 2 >2
Types of insulin regimen	Type and combination of insulin used obtained on the recruitment day and on the eighth visit obtained from medical records	Basal Premixed Basal-bolus

## Discussion

DMTAC service in primary public health clinics across Perak significantly improved glycaemic control (HbA1c and FBS levels) and BP compared with the baseline in this study, which is consistent with previous findings.^8,9,14,18^ However, whether LAIs confer better benefits in terms of the HbA1c level, FBS level, BP and LDL level than do SAIs after the completion of eight DMTAC visits remains unclear.

### Glycaemic control (HbAlc and FBS levels)

Our study observed a larger mean HbA1c level reduction in the SAI group than in the LAI group, although the mean difference was not significant. According to Silva et al., intensive training for self-titrating insulin doses combined with structured SMBG can significantly reduce the HbA1c level, but with no significant difference from less intensive training (p=0.051).^19^ In our study, the existence of IHD as co-morbidity in the LAI group (14.8%) compared with that in the SAI group (4.8%) yielded a larger mean HbA1c level reduction in the SAI group. This is possibly where a less stringent HbA1c level goal for patients with IHD is required, leading to a smaller mean HbA1c level reduction in accordance with the Clinical Practice Guidelines for T2DM in Malaysia. A more aggressive blood glucose level normalisation has no significant benefit on primary cardiovascular endpoints in patients with T2DM and only increases the risk of hypoglycaemia or weight gain.^14^

However, a higher percentage of Chinese in the LAI group skewed the mean HbA1c level difference to be insignificant. Ethnicity affects glycaemic control as well as complication profiles according to the 2019 National Health Morbidity Survey. Indians have been reported to have the highest prevalence, followed by the Malays and Chinese.^2^ The Chinese in the LAI group had a lower prevalence owing to less insulin resistance, which led to better glycaemic control with a larger mean HbA1c or FBS level reduction, than the other ethnicities.^20^

There is also a complex interaction among different factors, including the patient’s diet, individual motivation, self-care behaviour and knowledge for better glycaemic control.^19,20^ Of these, a dedicated self-care behaviour, including diet control, physical activity and medication adherence along with blood glucose monitoring, is a key factor.

### Blood pressure

DMTAC services significantly improved the DBP in both groups but did not significantly affect the SBP DBP elevation is easier to control by reducing arteriolar resistance to blood flow using current antihypertensive agents than SBP elevation in terms of reducing the arterial stiffness.^21^

Herein, the LAI group had a proportionately larger margin of mean DBP reduction than the SAI group. This is conflicting with several findings that BP control was achieved sooner in patients with SAIs.^22-24^ This difference could be attributed to the higher percentage of participants with cardiovascular comorbidities (IHD) in the LAI group than in the SAI group, affecting the management plan throughout the visits. The baseline DBP was also significantly higher in the LAI group. According to Tsujimoto et al. and Upadhya et al., a more intensive treatment approach is needed to achieve the target SBP and DBP goals to decrease the risk of subsequent cardiovascular events and death.^25,26^

### Cholesterol level

The LDL level did not significantly differ between the SAI and LAI groups (p=0.246). This result was expected, as LDL cholesterol levels are dependent on multifactorial causes. Dietary modifications, physical activity, reluctancy of dose titration by the patient and limited consultation time in changing behaviours towards achieving LDL level goals are challenging.^27,28^

### Strengths and limitations

Most prior studies examined only the effects of DMTAC services involving a structured educational programme with scheduled clinic visits on glycaemic control in patients with diabetes without severe hypoglycaemic events.^15,16^ These services usually require at least eight follow-up visits for each patient. To our knowledge, our study is the first to analyse the association between DMTAC appointment interval and glycaemic control. We identified the appropriateness of scheduled appointments (1–3 months) by analysing all registered DMTAC patients in Perak. Furthermore, we did not exclude DMTAC patients with hypoglycaemic events. Our findings can then be generalised to populations with T2DM registered with DMTACs in Perak.

Despite these strengths, the study also has several limitations. The existence of confounding factors, such as IHD and hypertension, was inevitable during matching at baseline. This might have contributed to the outcomes that were inconsistent with most previous findings.^3-7,16^ Nevertheless, the participants were grouped after matching based on the average duration of clinic visits for a total of eight visits to eliminate confounding factors, including the mean age and baseline anti-glycaemic regimen. In terms of internal validity, there was no control group employed in the study.

### Implications for clinical practice and research

In this study, glycaemic control, BP and LDL level did not significantly differ between the SAI and LAI groups. This finding is consistent with actual data in the practice of scheduling DMTAC appointments in Malaysia ranging from 1 to 3 months. This study can help pharmacists determine appropriate appointment intervals in public health clinics with a low manpower and high patient volume. Nevertheless, a higher frequency of visits is linked to improved treatment adherence and opportunities for medication intensification.^8,9,29,30^ Hence, an effective DMTAC service workflow can be generated by considering a proper follow-up appointment scheduling to improve glycaemic control. The care of patients with high-risk factors, such as hypoglycaemic events and co-morbidities, should not be compromised while scheduling appointments for closer monitoring during each clinic visit.^14,18,20,23^ Further prospective investigations into other confounding factors, which would most likely yield a more reliable outcome, are needed.

## Conclusion

Longer DMTAC appointment intervals had similar improvement in glycaemic controls, blood pressure and lipid profiles as compared to shorter appointment intervals. Longer intervals can be scheduled for lowerrisk patients to optimise the use of human resources and minimise costs.
